# Predicting the Release Mechanism of Amorphous Solid Dispersions: A Combination of Thermodynamic Modeling and In Silico Molecular Simulation

**DOI:** 10.3390/pharmaceutics16101292

**Published:** 2024-10-02

**Authors:** Stefanie Walter, Paulo G. M. Mileo, Mohammad Atif Faiz Afzal, Samuel O. Kyeremateng, Matthias Degenhardt, Andrea R. Browning, John C. Shelley

**Affiliations:** 1 AbbVie Deutschland GmbH & Co. KG, Small Molecule CMC Development, Knollstraße, 67061 Ludwigshafen am Rhein, Germany; stefanie.walter@abbvie.com (S.W.); matthias.degenhardt@abbvie.com (M.D.); 2 Materials Science, Schrödinger GmbH, Glücksteinallee 25, 68163 Mannheim, Germany; paulo.mileo@schrodinger.com; 3 Materials Science, Schrödinger LLC, Suite 1300, 101 SW Main Street, Portland, OR 97204, USA; atif.afzal@schrodinger.com (M.A.F.A.); andrea.browning@schrodinger.com (A.R.B.); 4 Life Science, Schrödinger LLC, Suite 1300, 101 SW Main Street, Portland, OR 97204, USA

**Keywords:** amorphous solid dispersion, release, phase behavior, liquid-liquid phase separation, PC-SAFT, coarse-grained modeling, dissipative particle dynamics, DPD

## Abstract

Background: During the dissolution of amorphous solid dispersion (ASD) formulations, the drug load (DL) often impacts the release mechanism and the occurrence of loss of release (LoR). The ASD/water interfacial gel layer and its specific phase behavior in connection with DL strongly dictate the release mechanism and LoR of ASDs, as reported in the literature. Thermodynamically driven liquid-liquid phase separation (LLPS) and/or drug crystallization at the interface are the key phase transformations that drive LoR. Methods: In this study, a combination of Perturbed-Chain Statistical Associating Fluid Theory (PC-SAFT) thermodynamic modeling and in silico molecular simulation was applied to investigate the release mechanism and the occurrence LoR of an ASD formulation consisting of ritonavir as the active pharmaceutical ingredient (API) and the polymer, polyvinylpyrrolidone-co-vinyl acetate (PVPVA64). A thermodynamically modeled ternary phase diagram of ritonavir (PVPVA64) and water was applied to predict DL-dependent LLPS in the ASD/water interfacial gel layer. Microscopic Erosion Time Testing (METT) was used to experimentally validate the phase diagram predictions. Additionally, in silico molecular simulation was applied to provide further insights into the phase separation, the release mechanism, and aggregation behavior on a molecular level. Results: Thermodynamic modeling, molecular simulation, and experimental results were consistent and complementary, providing evidence that ASD/water interactions and phase separation are essential factors driving the dissolution behavior and LoR at 40 wt% DL of the investigated ritonavir/PVPVA64 ASD system, consistent with previous studies. Conclusions: This study provides insights into the potential of blending thermodynamic modeling, molecular simulation, and experimental research to comprehensively understand ASD formulations. Such a combined approach can be leveraged as a computational framework to gain insights into the ASD dissolution mechanism, thereby facilitating in silico screening, designing, and optimization of formulations with the benefit of significantly reducing the number of experimental tests.

## 1. Introduction

Amorphous solid dispersion (ASDs) is a commonly used enabling approach to formulate drugs with low water solubility and limited bioavailability (BCS II and IV) [1,2,3]. To create an ASD, the crystalline drug is converted into an amorphous state and incorporated into an amorphous hydrophilic polymer matrix [4,5]. The incorporation of the amorphous drug into the polymer matrix leads to improved bioavailability due to enhanced dissolution characteristics and increased apparent solubility of the drug in aqueous medium [6,7,8,9]. However, high dissolution rates can lead to a supersaturated solution, and the drug may precipitate into the crystalline state or form amorphous drug-rich domains, impacting the ASD’s performance [10]. An ASD is only thermodynamically stable, i.e., the drug would never crystallize from the amorphous phase, as long as the drug concentration does not exceed its solubility in the polymer/water mixture [6,11,12]. Additionally, the phase transformation can be slowed down by the glassy state of the ASD since, below the glass transition, the molecular mobility of the system is reduced [13]. 

When developing an ASD formulation, high drug load (DL) is often preferred due to reducing pill burden and increasing patient compliance. However, a well-known drawback of the ASD formulation technique is the fact that the dissolution rate (drug release) decreases with increasing DL. The effect has been experimentally observed, extensively investigated, and discussed in the literature [14,15,16,17,18,19,20,21]. However, the underpinning thermodynamic principles of the phenomenon are less investigated and reported in the literature. Flory-Huggins theory often provides a thermodynamical description of these systems, with interaction parameters determined through experimental or computational methods. However, the intercomponent-specific interactions in ASDs, such as hydrogen bonds, are not considered [10]. On the other hand, the Perturbed-Chain Statistical Associating Fluid Theory (PC-SAFT) thermodynamic model, which considers specific intercomponent interactions, has been successfully applied to several ASD systems for phase behavior predictions [22,23,24,25]. For example, ASD formulations composed of the drug, ritonavir, and the polymer, polyvinylpyrrolidone-co-vinyl acetate (PVPVA64), were investigated by Krummnow et al. [22], whereby the ternary drug/polymer/water phase diagram was modeled with PC-SAFT to predict liquid-liquid phase separation (LLPS) [26]. Furthermore, using confocal Raman spectroscopy and differential scanning calorimetry, the authors quantitatively validated the water-induced LLPS predictions based on the modeled ternary phase diagram. The predicted LLPS phenomenon and its impact on the drug release rate with increasing DL corresponded well to the experimental findings of Yang et al. [14], Bochmann et al. [15], and Indulkar et al. [27].

Recently, Dohrn et al. [24] applied, for the first time, thermodynamic and glass transition modeling to provide more insights and a generalized explanation of the phase behavior at the ASD/water interfacial layer for fast- and slow- or non-crystallizing drugs. Based on modeled drug/polymer/water ternary phase diagrams, the authors predicted a priori and validated DL-dependent LLPS or recrystallization in the ASD/water interfacial gel layer, which subsequently dictates the ASD release mechanism [24,28]. However, a more thorough understanding of the mechanisms involved in the early and late stages of ASD dissolution additionally requires molecular-scale insights into the chemical processes at play in the ASD release. For instance, molecular simulation techniques have been successfully employed in elucidating drug/polymer intermolecular interactions, solubility parameters, and simulation of ASD formation and dissolution mechanisms [10,29,30,31,32,33,34,35]. Dissipative particle dynamics (DPD) simulations have been employed to understand the impact of polymer type and interfacial behavior of the ASDs during dissolution [10]. Molecular-level simulations provide information on the specific interactions that dominate a mixture and their contributions to the observed behavior [31]. For the complex phase behavior of ASDs with water, thermodynamic and molecular modeling can provide information that complements what is available from experiments [10]. Therefore, a combined approach leveraging molecular-level simulations and thermodynamic modeling can provide deeper insights into the complex phase behavior of ASDs during dissolution. Such a novel combined modeling approach is uncommon in the literature.

In this work, PC-SAFT thermodynamic modeling and DPD simulations are combined to understand the drug and polymer release mechanism and, independently, to predict in silico the DL-dependent loss of release (LoR) of ritonavir/PVPVA64 ASD. The modeling findings and in silico predictions were compared with experimental microscopic erosion time test (METT) images to provide a novel, comprehensive understanding of the dissolution mechanism of ritonavir/PVPVA64 ASDs.

## 2. Modeling, Materials, and Methods

### 2.1. Thermodynamic Modeling of Drug/Polymer/Water Phase Diagrams

#### 2.1.1. Solid-Liquid Equilibrium

The equilibrium between the pure solid (crystalline) phase, drug, and the liquid (amorphous) phase was considered for calculating the crystalline drug (here ritonavir) in water or polymer (here PVPVA64) and mixtures thereof according to Equation (1):

(1) $$x_{i}^{L}=\frac{1}{\gamma_{i}^{L}}exp\left[-\frac{\Delta h_{i}^{SL}}{RT}\left(1-\frac{T}{T_{i}^{SL}}\right)-\frac{\Delta c_{p,i}^{SL}}{R}\left(\ln\left(\frac{T_{i}^{SL}}{T}\right)-\frac{T_{i}^{SL}}{T}+1\right)\right]$$

where T is the temperature in K, R is the universal gas constant, and $x_{i}^{L}$ is the mole fraction of the component *i* in the liquid phase. The melting properties of the component i, namely the melting temperature $T_{i}^{SL}$, the melting enthalpy $\Delta h_{i}^{SL},$ and the difference between the solid and liquid heat capacities $\Delta c_{p,i}^{SL}$ of the drug, were taken from the literature (Table 1). The activity coefficient $\gamma_{i}^{L}$ of components i in the liquid phase accounts for deviations from an ideal mixture and was determined using PC-SAFT (Section 2.4). The polymer PVPVA64 can only be present in the liquid (amorphous) phase, as it neither evaporates nor crystallizes [36]. The drug is either amorphously dissolved in the liquid phase or present in a pure crystalline (solid phase) [37] or both. 

#### 2.1.2. Liquid-Liquid Equilibrium

In the case of liquid-liquid phase separation (LLPS), all three components, drug, polymer, and water, coexist in two phases, L1 and L2. Liquid-liquid phase separation was determined by simultaneously solving Equation (2) for each component *i* [22,37].

(2) $$x_{i}^{L1}\gamma_{i}^{L1}=x_{i}^{L2}\gamma_{i}^{L2}$$

$x_{i}^{L1}$ and $x_{i}^{L2}$ are the mole fractions of component *i* in phase L1 and phase L2, respectively. The calculated mole fractions were converted into mass fractions in the phase diagrams. The activity coefficients $\gamma_{i}^{L1}$ and $\gamma_{i}^{L2}$ component *i* in phase L1 and phase L2 account for the strong deviations in the interactions compared to an ideal mixture, which was determined with PC-SAFT [38].

#### 2.1.3. Glass-Transition

The glass transition temperature (*T*_g_) of drug/polymer/water mixtures was calculated using the Kwei equation [26] (Equation (3)).

(3) $$T_{g}=\frac{\sum_{i}K_{i\;}w_{i}T_{g,i}}{\sum_{i}K_{i\;}w_{i}}+\sum_{i\neq j}w_{i}w_{j}q_{ij}$$

where *w_i_* are the mass fractions of *i* equals polymer, drug, and water, respectively. *T*_g,*i*_ is the glass transition temperature of each pure component. Table 2 lists the *T*_g,*i*_ and true densities $\rho_{i}$ of the pure components, taken from the literature. The interaction parameters $q_{ij}$ were taken from the literature [22] (Table 3). The interaction parameters $K_{i\;}$ are calculated in the context of the polymer and were predicted using the Simha-Boyer rule [39] according to Equation (4):

(4) $$K_{i}=\frac{\rho_{polymer}T_{g,polymer}}{\rho_{i}T_{g,i}}$$

### 2.2. PC-SAFT

Activity coefficients used in this work were calculated using PC-SAFT [38] from the residual Helmholtz energy $a^{res}$ by summing up specific molecular contributions caused by repulsion (hard chain $a^{hc}$), attraction (dispersion $a^{disp}$), and association ($a^{assoc}$) according to Equation (5):

(5) $$a^{res}=a^{hc}+a^{disp}+a^{assoc}$$

Each molecule has a defined number of segments ($m_{i}^{seg}$), with a segment diameter, $\sigma_{i}$ and a dispersion energy parameter, ${(u}_{i}/k_{B})$. Hydrogen-bond-forming molecules are characterized by the association sites, $N_{i}^{assoc}$ the association-energy parameter $\left(\varepsilon^{A_{i}B_{i}}/k_{B}\right)$, and the association-volume parameter,$\;\kappa^{A_{i}B_{i}}$. $k_{B}$ is the Boltzmann constant. The pure-component parameters of ritonavir, PVPVA64, and water were taken from the literature (Table 4).

The combining rules of Berthelot [46] (Equation (6)) and Lorentz [47] (Equation (7)) were applied to determine the segment diameter $\sigma_{ij,}$ and the dispersion energy $u_{ij}$ in mixtures of components i and j:

(6) $$\sigma_{ij}=\frac{1}{2}\left(\sigma_{i}+\sigma_{j}\right)$$

(7) $$u_{ij}=\sqrt{u_{i}u_{j}}\;\left(1-k_{ij}\right)$$

To calculate the association energy and the association volume in mixtures of components i and j, the combining rules of Wolbach and Sandler [48] (Equations (8) and (9)) were used:

(8) $$\varepsilon^{A_{i}B_{j}}=\frac{1}{2}\left(\varepsilon^{A_{i}B_{i}}+\varepsilon^{A_{j}B_{j}}\right)$$

(9) $$\kappa^{A_{i}B_{j}}=\sqrt{\kappa^{A_{i}B_{i}}\kappa^{A_{j}B_{j}}}{\left(\frac{\sqrt{\sigma_{i}\sigma_{j}}}{\frac{1}{2}\left(\sigma_{i}+\sigma_{j}\right)}\right)}^{3}$$

The binary interaction parameter $k_{ij}$ corrects for deviations from the geometric mean of the dispersion energies of the pure components and might depend on temperature as expressed in Equation (10):

(10) $$k_{ij}=k_{ij,m}T+k_{ij,b}$$

Table 5 lists all interaction parameter’s coefficients $k_{ij,m}$ and $k_{ij,b}$ used in this work.

### 2.3. Molecular Simulation

#### 2.3.1. Dissipative Particle Dynamics and Its Parameterization

**Figure 1:**
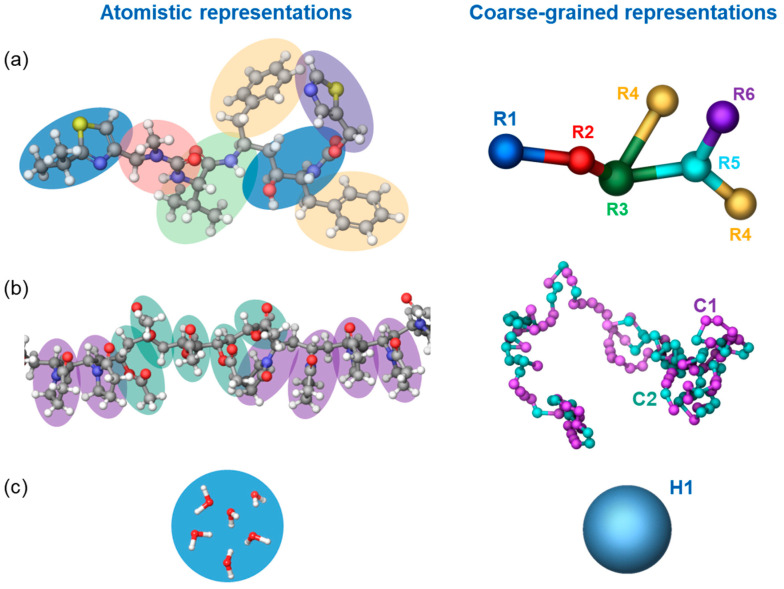
Grouping of atoms (on the left) into particles (on the right), employed for the coarse-grained representation of ritonavir (**a**), PVPVA64 (**b**), and water (**c**) molecules. Hydrogen, oxygen, carbon, sulfur, and nitrogen atoms are displayed as small white, red, gray, yellow, and blue balls, respectively.

Due to local inhomogeneities and the relatively long relaxation timescales involved in the dissolution process, its molecular-scale representation requires large system sizes and long simulation times. These challenges were mitigated by employing a coarse-grained simulation method: dissipative molecular dynamics (DPD). In DPD, the number of degrees of freedom of a system is reduced by grouping atoms into particles. These particles interact via pairwise additive non-bonded conservative, dissipative, and random terms, as detailed in previous work [10]. In addition to these non-bonded interactions, DPD particles interact with other nearby particles in the same molecule using harmonic bond and bond-angle potentials. The non-bond interaction between two particles depends on a single parameter, a_ij_, and is determined by the type of each particle. Each type of bond has a harmonic potential with two parameters: b_ij_, the equilibrium bond length, and $k_{ij}^{b}$ the force constant, representing the stiffness of the bond. Similarly, each type of bond angle has a harmonic potential with two parameters: θ_ij_, the equilibrium angle, and $k_{ij}^{\theta }$ the force constant, representing the stiffness of the angle. Further details on our DPD methodology can be found in previous work [10]. The values for these parameters were determined using the automatic parametrization toolbox “Coarse-Grained Forcefield Builder” available in Schrödinger Materials Science Suite [49], with the exception of the parameters for the PVPVA polymer, which were previously determined based on the polymer dissolution pattern in water. In this procedure, multiple DPD simulation cycles are run in which the force field parameters are iteratively adjusted in order to match structural features determined from reference all-atom (AA) molecular dynamics (MD) simulations. DPD simulations were carried out at a constant reduced density of 3 in the canonical (constant number of particles, volume, and temperature, NVT) ensemble and with a cutoff radius of 8.15 Å. Figure 1 depicts the mapping of atoms onto DPD particles used in our simulations. Each particle represents connected atoms defined to avoid splitting up functional groups and to have a volume similar to all the other particles. Their corresponding masses were assigned by the sum of each of their constitutive atoms. Each PVPVA64 polymer molecule consisted of 100 vinyl-pyrrolidone (DPD particle type C1) and 86 vinyl acetate (DPD particle type C2) monomers in a random order along the chain. This corresponds to approximately one-third of the average molecular weight of polymer samples used in the experimental studies, a size that was previously demonstrated to be sufficiently large enough to capture the polymer’s dissolution behavior [10]. Ritonavir molecules were each represented by six DPD sites (R1—R6), while a group of six neighboring water molecules was represented by a single DPD bead (H1).

The reference AA MD system contained an 80:20 wt% ratio of water and solute. Additionally, the solute proportion consisted of 12:88 wt% ritonavir in PVPVA64. To prepare this simulation system, molecules were initially placed into an expanded simulation cell with an initial density of 0.5 g/cm^3^. As part of this process, non-solvent molecules were constructed using a random walk scheme (tangled chain) as implemented in the disordered system builder panel of the Maestro Materials suite. Non-bonded interactions were modeled by a sum of Coulombic and 6–12 Lennard-Jones (LJ) potentials (the latter represents a sum combination of excluded volume and dispersion interactions). These potentials, as well as valence interaction terms, were parameterized according to the OPLS4 forcefield [50]. Dispersive interactions were limited to a maximum interatomic distance of 9 Å with a long-range correction term for interactions beyond that distance, while electrostatic interactions were calculated using the U-series treatment [51]. The equilibration and following production simulations were carried out in the isothermal-isobaric (constant number of particles, pressure, and temperature—NPT) ensemble at 310 K and 1 bar for 100 ns using the Nose-Hoover chain thermostat [52] and the Martyna-Tobias-Klein barostat [53], with respective relaxation constants of 1 and 2 ps and a timestep of 2 fs for most interactions except for the long-range electrostatic interactions for which the time step was 6 fs.

DPD simulations were carried out in the NVT ensemble at 310 K with a 30 fs timestep in Desmond [54,55] using the formalism developed by Groot and Warren [56]. The systems’ construction utilized the Disordered System Builder in Schrödinger Materials Science Suite, with the particle assignment and forcefield derivation obtained employing the Maestro Materials and Materials Coarse Grain [49] software. Prior to each DPD simulation, the systems were equilibrated with 100 ps Brownian dynamics at 10 K, 100 ps of Langevin dynamics in the NVT ensemble at 10 K, and 100 ps Langevin dynamics at 300 K.

#### 2.3.2. DPD Simulations of Early-Stage Dissolution

An anhydrous drug/polymer mixture was first built and relaxed in a cubic system. A copy of this was placed at each end of a long, narrow orthorhombic simulation box with a square cross-section. The much larger central region of this simulation box was then filled with water. The water/solid ratio in these systems (ca. 4.2–3.0 g/g) is considerably smaller in comparison with their experimental counterparts, leading to saturated solutions after minimal (<1%) drug dissolution. Nevertheless, our models already employ a relatively large number of particles, and using a larger number of water molecules would be computationally prohibitive. To imitate a much larger aqueous solution in which drug molecules can diffuse into remote aqueous regions, each particle of the molecules that had diffused sufficiently far from the ASD region was converted into water particles. This region (converting region) was kept at the center of the aqueous region and comprised 58% of the system’s volume. A similar methodology of ASD construction was employed as in previous work [10] to study the aqueous dissolution mechanisms of PVPVA64 and Soluplus^®^ polymers in the absence and presence of drugs. Due to the typically long timescales associated with the simulation of dissolution processes, a 12.5% increase in the mutual water particles’ repulsive parameters was employed in order to increase the solubility of both drug and polymer into the water medium, thereby accelerating the overall dissolution. DPD simulations were then carried out in a series of cycles, each consisting of a 200 ns simulation followed by the conversion of the drug molecules within the converting region using a custom script. This was carried out for 12 cycles with an overall simulation time of 2.4 μs.

#### 2.3.3. DPD Simulations of Late-Stage Dissolution

For each late-stage simulation, the solutes, the polymer, and the drug comprised a total of 2.5 wt% of the system, with water comprising the rest of the system. In late-stage dissolution simulations, the polymer and drug molecules are not pre-equilibrated in a separate zone before being exposed to water. Instead, the system was constructed by randomly placing molecules of all types in the starting structure prior to equilibration and spontaneous association into aggregates. The same concentrations of drug molecules relative to the polymer as in the early-stage simulations were employed. Thereafter, DPD simulations were carried out in the NVT ensemble for 2.0 μs at 310 K. As in the previous section, 10 replicates of each simulating system were employed.

### 2.4. Materials and Experimental Methods

Ritonavir (Form II) was obtained from AbbVie Inc. (Chicago, IL, USA). Vinylpyrrolidone-vinyl acetate copolymer (PVPVA64, Kollidon^®^ VA 64) was purchased from BASF SE (Ludwigshafen, Germany).

#### 2.4.1. Preparation of ASD Discs

The ASDs used in the METT experiments were based on cryo-milled physical blends, which were melted and shaped using a vacuum compression molding (VCM) tool (MeltPrep GmbH, Graz, Austria) as described in previous works [15,24]. For the cryo-milling, approximately 1 g of each drug/PVPVA64 physical blend was loaded into 10 mL stainless steel chambers with a 15 mm stainless steel ball. The chambers were put into liquid nitrogen and subsequently milled in an MM400 device (Retsch GmbH, Haan, Germany) at 30 Hz for 30 s. To prepare disc-shaped ASDs, approximately 50 mg of the cryo-milled blend was loaded into the VCM tool with a 10 mm diameter disc geometry and then annealed for 20 min. Annealing temperatures of 140–160 °C were applied. The absence of crystalline residuals in the prepared discs was confirmed using polarized light microscopy [24]. ASD discs with DLs 1 wt%, 5 wt%, 15 wt%, 20 wt%, and 40 wt% were prepared.

#### 2.4.2. Microscopic Erosion Time Test (METT)

The METT test was performed with a Keyence VH-X digital microscope (Keyence Deutschland GmbH, Neu-Isenburg, Germany) with a mounted copper plate tempered at 37 °C by a Thermostat Haake A10 (Thermo Fisher Scientific, Karlsruhe, Germany) [24]. On a glass slide located on the copper plate, an ASD disc was placed and then covered with a coverslip. 0.5 mL of pre-heated, degassed, demineralized water was introduced to the circumference of the ASD disc, and the edges of the coverslip were sealed with nail polish to prevent water loss. Images in the course of the ASD erosion into the water were taken every 10 min over a 60 min period. More details on the METT equipment setup can be found in the publication by Bochmann et al. [15].

## 3. Results and Discussion

### 3.1. Thermodynamic Modeling of the ASD/Water Interface

**Figure 2:**
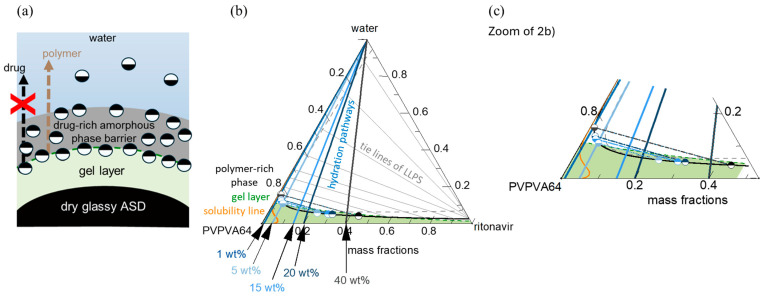
(**a**) Schematic of ASD/water interfacial layers depicting a dry glassy ASD core, hydrated gel layer, and hydrophobic drug-rich amorphous barrier formation triggered by LLPS. (**b**) Ternary phase diagram of ritonavir/PVPVA64/water system at 37 °C, and (**c**) zoom-in showing the solubility line (orange), binodal line (black), or LLPS boundary, tie lines (solid-gray), spinodal line (dashed-gray), and the glass transition line (dashed-green). The colored lines running towards the apex indicate the hydration pathways through the ASD/water interface into the bulk, starting from the dry ASD towards increasing water concentration for 1 wt%, 5 wt%, 15 wt%, 20 wt%, and 40 wt% DLs. The lower and upper half-filled circle symbols schematically refer to the corresponding polymer-rich (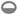) and drug-rich (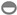) phases, respectively, after LLPS at *eGT*.

A previous thermodynamic modeling approach was utilized to predict the interfacial behavior between PVPVA64-based ASDs and water [24]. It considers various interconnected processes, such as water ingression, LLPS, and crystallization, which collectively determine the interfacial behavior between the ASD and water and, subsequently, the dissolution performance of the formulation [24]. During dissolution, successive layers with increasing water concentration from the core to the surface of the ASD are formed as water ingresses the formulation. These layers, schematically depicted in Figure 2a, are mainly a water-free core layer (dry glassy core), a hydrated but glassy layer (gel layer) below the “escape glass transition (*eGT*)”, and a sufficiently hydrated layer above *eGT*, which erodes into the bulk aqueous medium as previously described by Dohrn et al. [24]. For a slow crystallizing drug, LLPS can occur within the hydrated layers, leading to the formation of a hydrophobic drug-rich amorphous barrier at the interface. The interplay between the different levels of hydration in the layers and potential phase transformations can be understood by tracing the hydration pathway for a specific drug-loaded ASD (DL-ASD) through a ternary drug/polymer/water phase diagram. The modeled ternary phase diagram for the ritonavir/PVPVA64/water system and the hydration pathways for the investigated ASD DLs are shown in Figure 2b,c.

The ternary phase diagram shows the solubility of the drug in the PVPVA64/water mixture (orange line) and an LLPS region defined by the black binodal line, which slightly overlaps with the glass region (area below the dashed-green glass transition line). Within the drug-supersaturated region, thermodynamically driven phase transformation will occur. Depending on the concentration of the ternary mixture, the driving force(s) for equilibrium determines what phase changes might occur, e.g., either ritonavir crystallization or LLPS or both [24]. However, the system’s mobility is reduced at concentrations below the glass transition line, which can kinetically hinder phase changes. Since amorphous ritonavir is a known slow re-crystallizer in solid or aqueous environments, the driving force for crystallization is low compared to LLPS during the ASD dissolution [22,27]. Hence, subsequent discussions will focus mainly on LLPS during dissolution. Considering the five different DLs depicted in Figure 2b, on contact with water, the surface of the dry ASD immediately starts absorbing water along the hydration pathways, and the first hydrated gel layer is formed at the ASD/water interface. The absorbed water molecules act as plasticizers and decrease the formed gel layer’s glass transition temperature (*T*_g_) compared to the dry ASD core. As the water concentration in the gel layer increases and the *T*_g_ continues to drop and approaches *eGT*, at which point the *T*_g_ is equivalent to the temperature of the dissolution medium (37 °C), the gel layer switches to a more fluid (water-like) layer, thus, initiating the release of the ASD from the interface. The *eGT* is indicated by the dashed green glass transition line in Figure 2b. According to the hydration pathways in Figure 2b,c, except for the 1 wt% DL ASD, all the investigated DLs will encounter the binodal line at *eGT* and, thus, phase separate into an amorphous drug-rich (ritonavir-rich) and a polymer-rich (PVPVA64-rich) phase at the ASD/water interface. The initiated phase separation could result in the enrichment of hydrophobic amorphous drug at the interface if the hydrophilic polymer-rich phase is preferentially released into the aqueous medium, as schematically shown in Figure 2a [24,57]. The enrichment can lead to the formation of a hydrophobic drug-rich amorphous barrier at the interface and hinder further ingress of water into the ASD, thereby slowing down or preventing further dissolution of the formulation. This phenomenon whereby the drug-rich amorphous phase enriches the ASD surface while the polymer-rich phase preferentially erodes into the water is commonly referred to as loss-of-congruency (LoC) [58,59] and leads to a Type II loss-of-release (LoR) mechanism [24].

To assess the impact of LLPS on the ASD/water interfacial behavior during hydration, the thermodynamic endpoint composition and relative amounts of both polymer-rich and drug-rich phases at *eGT* were calculated by following the LLPS tie lines in Figure 2b and applying the lever rule. The results of the calculations for mass fractions, composition of polymer-rich and drug-rich phases, and resulting *T*_g_ are presented in Table 6. As previously mentioned, the 1 wt% DL ASD hydration pathway intersects the binodal line above *eGT* (see Figure 2); hence, phase separation is predicted to occur in the bulk water away from the ASD/water interface. Thus, the 1 wt% DL ASD hydration pathway is not considered in Table 6.

The results in Table 6 (phase mass fraction column) show that as the DL increases from 5 wt% to 40 wt%, the mass fraction of the polymer-rich phase decreases while that of the drug-rich phase increases, whereby the drug-rich phase reverses from the minor phase to major phase at 20 wt% and 40 wt% DLs. Furthermore, the mass fraction of the polymer in the polymer-rich phase at the thermodynamic end points of the LLPS (depicted in Figure 2b,c as the equilibrium endpoint of the binodal at the *eGT*) is nearly unchanged for the 5–40 wt% DL. Although the mass of the polymer is unchanging, the mass fraction of drug and water in the polymer-rich phase decreases and increases, respectively, with increasing DL. Hence, the *T*_g_ of the polymer-rich phase decreases significantly with increasing DL due to the increasing plasticization effect of water.

In contrast, the mass fraction of the hydrophobic drug in the drug-rich phase increased proportionally with the DL (Table 6, phase composition at the *eGT* binodal column). Thus, based on the hydration pathways from the ternary phase diagram and applying the lever rule, the LLPS formed drug-rich phase at the interfacial layer simultaneously increases in relative amount and drug concentration as DL increases. However, the drug-rich phase is not anticipated to passivate or immobilize the interfacial gel layer at 5 wt% and 15 wt% DL because it is a minor phase (Table 6, phase mass fraction column), and its *T*_g_ is not significantly different compared to the temperature of the aqueous phase (37 °C). This can be quite different for ASD formulated with a high *T*_g_ drug such as venetoclax, whereby the minor drug-rich phase can suddenly passivate the interfacial gel layer upon LLPS because the *T*_g_ of the drug-rich phase is extremely higher than the temperature of the aqueous phase [24]. Thus, for the ritonavir/PVPVA64 ASD with DLs up to 15 wt%, the polymer-rich phase, being the major phase upon LLPS, is expected to control the release behavior at the interfacial gel layer. Conversely, at 20 wt% DL, the drug-rich phase begins to dominate the interfacial gel layer, especially at 40 wt% DL when it excessively forms the major phase. Under such circumstances, the excessively high amount of the hydrophobic drug-rich phase at the interface can build an amorphous drug-rich barrier (passivation) as the hydrophilic polymer-rich phase erodes into the water preferentially.

**Figure 3:**
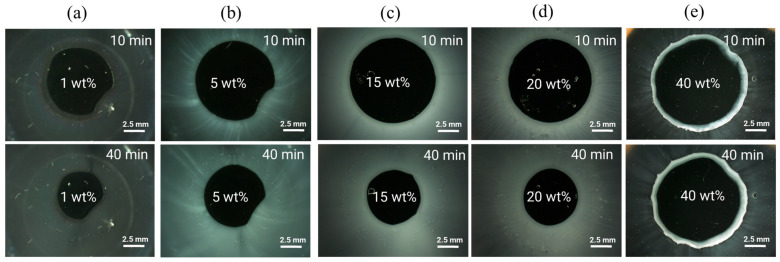
METT images at 37 °C of (**a**) 1 wt%, (**b**) 5 wt%, (**c**) 15 wt%, (**d**) 20 wt%, and (**e**) 40 wt% ritonavir-DL ASD discs after 10 and 40 min.

To verify the predicted ASD/water interfacial gel layer behavior, the dissolution of ritonavir/PVPVA64 ASDs with 1 wt%, 5 wt%, 15 wt%, 20 wt%, and 40 wt% DL manufactured with the VCM tool was monitored via METT experiments. METT is a very effective way of monitoring gel layer formation because hydrodynamic effects are absent compared to typical dissolution tests that involve stirring or agitation. Figure 3 shows the microscopy images of the eroding ASD discs after 10 and 40 min.

As seen across the images, except for the 1 wt% DL ASD, the 5–40 wt% DL ASDs erode into the bulk aqueous phase as fine white dispersions emanating directly from the interface between the dark ASD discs and the aqueous phase. The ASD discs appear dark due to strong light scattering caused by the white drug-rich phase droplets formed upon LLPS. Noticeably, for the 1 wt% DL ASD, the dispersion does not emanate immediately from the ASD/water interface but instead builds up in the aqueous phase away from the interface as predicted via the hydration pathway that LLPS will occur above *eGT*. The 5–40 wt% DLs build up the drug-rich phase dispersions (seen in Figure 3 as white foggy corona) at both the ASD/interface and in the aqueous phase due to the predicted LLPS at both *eGT* and beyond based on the hydration pathways in Figure 2b. Interestingly, it can be observed that for the 40 wt% DL ASD, a visible drug-rich gel layer forms at the ASD/water interface, which persists even after 40 min. This is attributable to the fact that, upon LLPS at *eGT*, the gel layer is predominantly composed of a drug-rich phase (0.87 mass fraction, ref. Table 6), as predicted. As expected, the high-content hydrophobic drug-rich phase passivates the gel layer and slows down or prevents further dissolution of the 40 wt% DL ASD; the disc size remained nearly unchanged after 40 min. Indulkar et al. [27] through fluorescence microscopy and FT-IR spectroscopy investigations found that the passivated gel layer formed by the 40 wt% DL ASD is composed mainly of a hydrophobic ritonavir-rich phase. Overall, the release mechanism predicted by thermodynamic modeling and confirmed by the METT experiments agrees very well with the reported release behavior of ritonavir/PVPVA64 ASDs, where LoR was observed at 40 wt% DL by Indulkar et al. [27], Bochmann et al. [15], and Krummnow et al. [22].

### 3.2. Molecular Simulations

**Figure 4:**
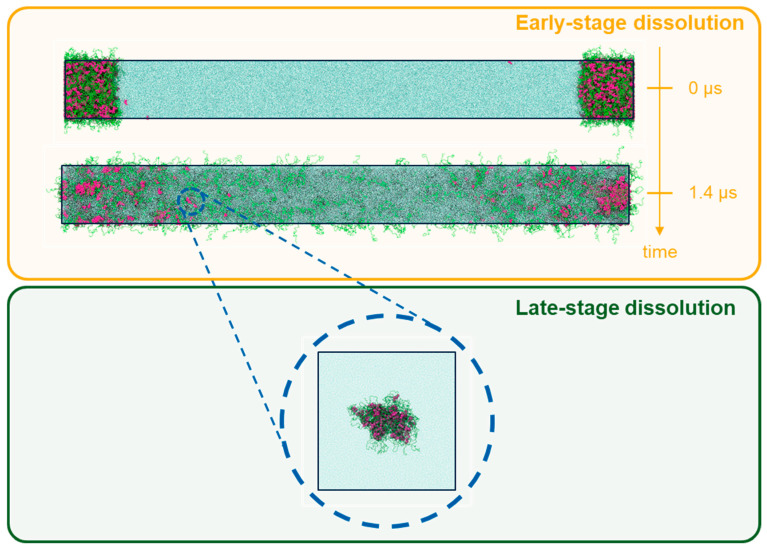
Illustration of the two types of simulation employed in this work. Early-stage dissolution (top), modeled by a periodic box containing an ASD formed by polymer (green tubes) and ritonavir (red spheres) in contact with an aqueous medium (blue dots), and late-stage dissolution (bottom), represented by a periodic box containing small self-assembled drug/polymer pockets enveloped by water.

In order to better understand the intricacies of the ASD dissolution process, dissipative particle dynamics simulations were carried out, as described in Section 2.3. These simulations were designed to study the ASD dissolution processes in two stages: early-stage dissolution, in which models of the ASDs have their initial outer layer surfaces exposed to an aqueous environment, and late-stage dissolution, in which the focus was to investigate the ritonavir-PVPVA64 and ritonavir-water interactions after drug and polymer have emanated from the ASD and are located in an aqueous environment. In Figure 4, a schematic representation of both simulation strategies is displayed.

**Figure 5:**
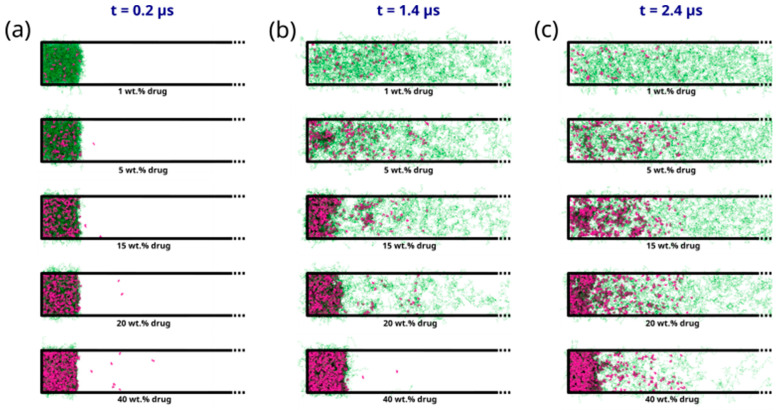
Snapshots displaying the evolution of the PVPVA64 (in green) and ritonavir (in purple) from ASD models with DL ranging from 1 wt% to 40 wt% obtained from early-stage dissolution molecular simulations at times of (**a**) 200 ns, (**b**) 1.4 μs, and (**c**) 2.4 μs. Only half of the simulation cells are displayed to better focus on the phenomena at the ASD/water interface. The full snapshots are provided in the supplementary information. Water molecules are omitted for clarity.

**Figure 6:**
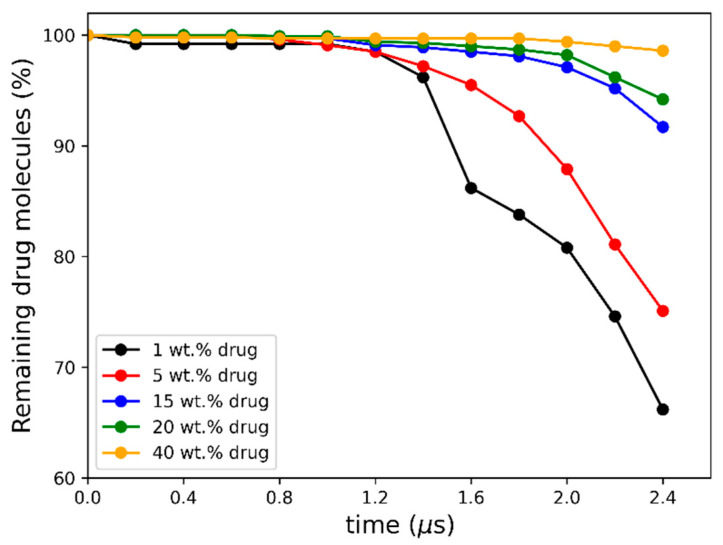
Percentage of non-converted drug molecules remaining in the simulation box after each 200 ns conversion cycle.

As seen in Figure 5a–c, the early molecular modeling simulation results show a clear dependence on the ASD DL. For 1 wt% DL, a quick disaggregation of the polymeric matrix in the ASD is observed together with the release of isolated drug molecules into the aqueous phase. A thorough polymer disaggregation is still observed at 5 wt%; however, the aggregation of the drug molecules into drug-rich phase clusters released into the aqueous phase can be noted. This observation is akin to LLPS into drug-rich and polymer-rich phases in the gel layer as the ASD is released into the aqueous phase, predicted by the thermodynamic modeling (Figure 2b and Table 6) and also observed in the METT image in Figure 3b. Interestingly, these drug clusters seem to be closely associated with the solvated polymer. These interactions will be analyzed in more detail later. As the DL further increases (15–20 wt%), the size and amount of the drug-rich phase clusters tend to increase, and the polymer matrix no longer fully disaggregates at the end of the 2.4 μs of simulation, indicating the dissolution kinetics of the ASD are slowing down. At this point, the ASD shows a fully solvated polymer phase with increased drug-rich phase clusters, which form the main aggregate. At an even higher DL (40 wt%), interestingly, the drug-rich phase clustering becomes so pronounced that only a smaller percentage of polymer and drug molecules appear to leave the ASD. This agrees very well with the predicted LLPS phase behavior based on the thermodynamic modeling (Table 6), which indicates that the ritonavir-rich phase excessively forms the major phase and is confirmed by the METT image in Figure 3e. The observed trend of decreasing relative drug dissolution with increasing DL is easily summarized by computing the percentage of molecules remaining in the simulation box (cf. Figure 6). In Figure 6, it is evident that the dissolution of the drug molecules slows down with increasing DL of the ASD, and a negligible amount of the drug is dissolved at 40% DL. This dissolution dynamics is highly comparable to the experimental results reported by Krummnow et al. [22] and Indulkar et al. [27]. It also agrees with the thermodynamic modeling results from the previous section, not only in terms of the formation of drug- and polymer-rich phases but also in terms of the DL required to shift the relevance of the drug-rich phase with respect to the dissolution behavior of ritonavir/PVPVA64 ASD.

In addition to the dependence of the ASD dissolution behavior on DL, the results in Figure 5 can also be correlated to the different interfacial ASD zones formed during the dissolution process, which has already been discussed in the thermodynamic modeling section. In direct correlation to the scheme shown in Figure 2a, the formation of different zones from the bulk aqueous phase to the dry ASD phase can also be observed from the MD simulations. This is particularly well noticeable in the intermediate DLs (e.g., 15–20 wt%) at 1.4 μs of the simulation (cf. Figure 5b). It is noteworthy that the partial solubilization of the polymer leads to the formation of a phase containing solubilized polymer with drug-rich phase clusters close to the ASD and the aqueous phase. This agrees with the predicted LLPS formed at the ASD/water interface (gel layer), as suggested by Figure 2b. Focusing on the region close to the core ASD, it is also noticeable that the polymer is less solubilized than in the previous zone and that the drug molecules start to form drug-rich clusters before being carried away into the bulk aqueous phase. This also agrees with the suggested ASD/water interface hydration mechanism in Figure 2b. It should, however, be mentioned that although the effect or impact of the glass transition described in the thermodynamic modeling section cannot be captured in the MD simulations, the formation of the drug-rich phase and its consequential impact on the dissolution behavior is in very good alignment with the thermodynamic modeling predictions.

**Figure 7:**
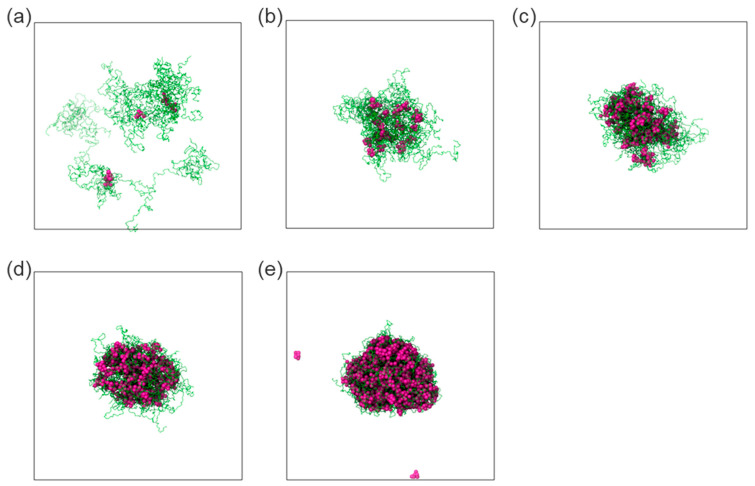
Snapshots displaying the associations of the polymer (in green) and drug (in purple) within water with DL of 1 wt% (**a**), 5 wt% (**b**), 15 wt% (**c**), 20 wt% (**d**), and 40 wt% (**e**), as obtained at the end of late-stage dissolution molecular simulations. Water molecules were omitted for clarity.

To model the late-stage dissolution process, the sizes of molecular aggregates formed by the early-stage dissolution of the ASD model at intermediate concentrations (5–15 wt%) were analyzed. The analyses revealed aggregates with radii of gyration ca. 50 Å (Figure S1). Considering this information, drug/polymer systems were set up with aggregates of similar sizes for the late-stage dissolution simulations. Compared to the previous early-stage simulations, the self-assembly equilibrium of drug and polymer molecules was more rapidly achieved within a few hundred nanoseconds. In these systems, it was observed that at very low drug DLs (1–5 wt%), drug molecules are surrounded by loose polymer chains and tend to associate with each other for isolated clusters (cf. Figure 7a,b).

**Figure 8:**
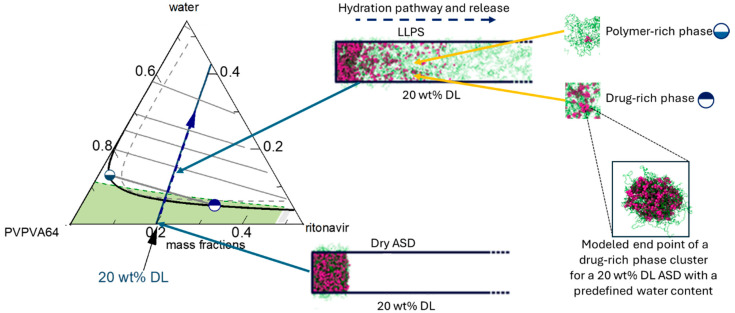
Zoom-in of the PVPVA64 corner of the ritonavir/PVPVA64/water ternary showing the hydration pathway (blue line) of 20 wt% DL ASD through the ASD/water interface into the water, starting from the dry ASD towards increasing water concentration. The half-filled circle symbols schematically correspond to the polymer-rich (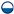) and drug-rich (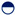) phases after LLPS at the interface. A schematic connection between the hydration pathway and the MD dissolution simulation of 20 wt% DL ASD is shown on the right.

As the DL increases (15–40 wt%), a tendency of the drug molecules to form consolidated clusters intertwined with polymer chains arises (cf. Figure 7c–e). It suggests again that, at low DL, the polymer is the dominant component driving the clustering behavior, while at intermediate and high DLs, the drug is the dominant component driving the clustering behavior. This observation is analogous to the early-stage MD modeling (Figure 5) and the thermodynamic calculations, which indicate that the drug-rich phase is the major phase at intermediate and high DLs (Table 6), as exemplified in Figure 8 for 20 wt% DL ASD. Figure 8 provides an overview of how the modeled thermodynamic phase diagram and MD simulations of the early- and late-stage dissolution are connected. Having compared the MD dissolution simulations to the thermodynamic modeling predictions and METT experiments, the focus here is shifted to a more in-depth analysis of the clusters in the late stage.

**Figure 9:**
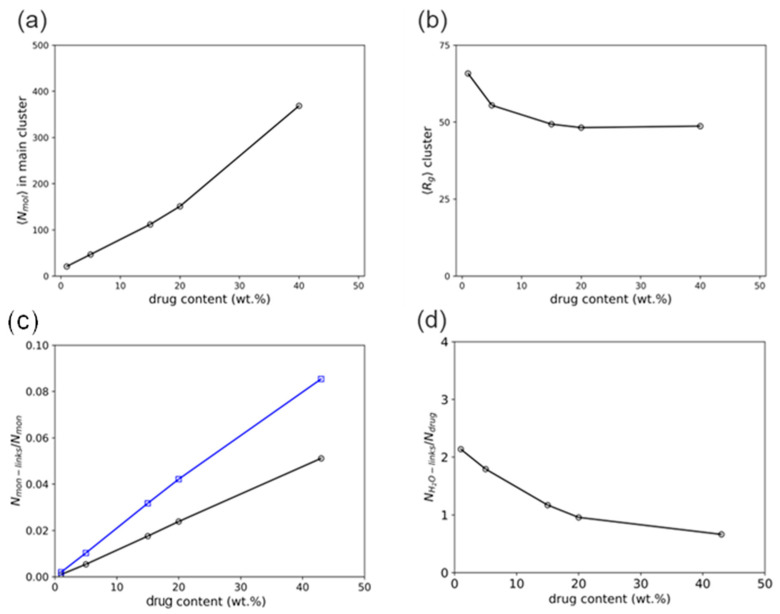
Series of analyses obtained from late-stage molecular simulations as functions of the DL: average number of drug and polymer molecules in the main self-assembled cluster, *N*_mol_ (**a**), average radius of gyration of said cluster, *R*_g_ (**b**), average number of close contacts between drug and polymer monomer particles per monomer, *N*_mon-links_/*N*_mon_ (**c**), and average number of close contacts between drug and water particles per drug molecule, *N*_H2O-links_/*N*_drug_ (**d**).

The number of molecules in the cluster, *N*_mol_ (Figure 9a), and cluster compactness (Figure 9b), which is reflected in the radius of gyration, *R*_g_, show a strong dependence on DL. It can be observed that as DL increases, *N*_mol_ also increases while *R*_g_ decreases and levels off at 20 wt% DL and above. This suggests that the cluster becomes increasingly compact with increasing DL due to the increasing association of the drug molecules, and the association phenomenon dominates above 20 wt%. From the thermodynamic calculations in Table 6, at 20 wt% DL and above, the drug-rich phase assumes the major phase after LLPS (Figure 8), which aligns well with the DL-dependent behavior of the clusters.

An analysis of the drug and polymer radial distribution on the main polymer/drug agglomerate (Figure S2) in the late dissolution stage revealed that the polymer chains at 15–20 wt% DL are closely intertwined with the drug molecules. At the highest concentration studied (40 wt%), the drug molecules are in full association with the polymer backbone instead of forming separate drug-rich zones, as observed during the early-stage simulations. This result suggests that the agglomeration of the drug into separate zones is probably a result of a collective association of the drug molecules at larger geometrical scales similar to the early-stage simulations.

To better understand the mutual association between drug and polymer, the interactions of the drug molecules and the polymer’s monomers were tracked in terms of the average number of close contacts, “links”, defined with a maximum 4 Å threshold between the drug and monomeric particles per monomer. Figure 9c shows that the drug molecules interact more frequently with the vinyl-pyrrolidone monomers than with the vinyl-acetate monomers. It is also noteworthy that the gap between these interactions increases with the DL, possibly as a consequence of the interaction of multiple molecules with the vinyl-pyrrolidone sites when in higher drug presence. Similarly, the interaction between ritonavir and water molecules was tracked with a similar metric, defined as the number of “binds” within an 8 Å threshold between water and drug particles per number of drug molecules. As seen in Figure 9d, the number of contacts of each molecule of drug with water monotonically decreases from ca. 2 water particles per drug molecule at the lowest DL (1 wt%) and levels off to less than ca. 1 water particle per drug molecule at the highest at 20 wt% DL and above. This can be directly correlated to the lesser exposure of drug molecules and hydrophobic interactions within the main cluster with the increase in the cluster size and the subsequent shielding of these molecules by the polymer occurring at high DL.

## 4. Conclusions and Outlook

The drug load-dependent release mechanism of ritonavir/PVPVA64 ASD was predicted independently by thermodynamic modeling of the ritonavir/PVPVA64/water ternary phase diagram using PC-SAFT and molecular simulations. The modeled ternary phase diagram, combined with the modeled glass transition using the Kwei equation, provided insights into liquid-liquid phase separation (LLPS) at the ASD/water interface. By applying the lever rule in the binodal region of the phase diagram at “escape glass transition” (*eGT*), the relative amounts of the phases upon LLPS in the ASD/water interfacial gel layer were calculated. Computations indicated that at low to moderate drug loading (5–15 wt%), PVPVA64 formed the major phase (polymer-rich phase), while ritronavir formed the minor phase (drug-rich phase). At high drug loading (20–40 wt%), this reversed. The composition and glass transition temperature, *T*_g_, of each phase were computed to predict the DL-dependent loss of release (LoR) Type II mechanism (formation of a drug-rich phase barrier in the gel layer at 40 wt% DL). The prediction was experimentally validated by monitoring the release behavior of ritonavir/PVPVA64 ASDs with 1–40 wt% DL using the microscopic erosion time test (METT). The predicted release behavior of the ASDs also aligned very well with previous findings by Indulkar et al. [27], Bochmann et al. [15], Shi et al. [21], and Krummnow et al. [22].

Molecular simulations offered additional insights into the dissolution behavior of the ASDs at the molecular level. The simulations revealed that, at the early stages of the ASD hydration, the drug molecules distinctively aggregate into drug-rich phase clusters for the high DL ASDs, delaying erosion and drug release considerably at 40 wt% DL. Later stage simulations of the dissolution revealed detailed interactions within the drug-rich clusters and with the surrounding water, indicating that dehydration and the stabilization of clusters are augmented with increasing DL. These nanoscale observations matched the results from thermodynamic modeling and METT experiments, providing comprehensive support for the overall mechanistic understanding of ASD dissolution.

Overall, this study demonstrates that the complementarity information gained from thermodynamic modeling, molecular simulation, and experiment provides a valuable and more complete understanding of the release mechanism of ritonavir/PVPVA64-based ASDs. Molecular-level interactions and their impact on ASD dissolution and aggregation behavior found in this study indicate that structure-function relationships can be determined by thermodynamic modeling and molecular simulation to support formulation design, thereby reducing the amount of trial-and-error sampling. Thus, a combined computational/experimental strategy can potentially support the identification of maximal DL as a possible starting point for further ASD formulation design and development.

## Figures and Tables

**Table 1:** Pure-ritonavir melting temperature, melting enthalpy, and the difference between solid and liquid heat capacity.

Component i	$T_{i}^{SL}/K$	$\Delta h_{i}^{SL}/kJ\;{mol}^{-1}$	$\Delta c_{p,i}^{SL}/J\;{mol}^{-1}K^{-1}$
ritonavir [37]	398.15	63.15	224

**Table 2:** Pure-component densities and glass transition temperatures used in this work.

Component i	$\rho_{i}/kg\;m^{-3}$	$T_{g,i}/K$
ritonavir	1151 [37]	323.5 [37]
PVPVA64	1190 [40]	384.15 [41]
water	1000 [42]	138.00 [43]

**Table 3:** Interaction parameters $q_{ij}$ of the binary mixtures of ritonavir, PVPVA64, and water used in this work.

Mixture	$q_{ij}$/K
ritonavir/PVPVA64	−18.83 [22]
ritonavir/water	−304.87 [22]
PVPVA64/water	34.82 [22]

**Table 4:** PC-SAFT pure-component parameters of the components investigated in this work.

Component i	$M_{i}/g\;{mol}^{-1}$	$m_{i}^{seg}M_{i}^{-1}/mol\;g^{-1}$	$\sigma_{i}/Å$	$u_{i}k_{B}^{-1}/K$	$\varepsilon^{A_{i}B_{i}}k_{B}^{-1}/K$	$\kappa^{A_{i}B_{i}}$	$N_{i}^{assoc}$
ritonavir [37]	721	0.0220	3.900	305.787	1041.0	0.02	4/4
PVPVA64 [44]	65000	0.0372	2.947	205.271	0	0.02	653/653
water [45]	18.015	0.0669	$\sigma_{water}$ *	353.950	2425.7	0.0451	1/1

* $\sigma_{water}=2.7927+10.11exp(-0.01755T/K)-1.417exp(-0.01146T/K)$.

**Table 5:** PC-SAFT interaction parameters for ritonavir, PVPVA64, and water mixtures.

Mixture	$k_{ij,m}/K^{-1}$	$k_{ij,b}$
ritonavir/PVPVA64 [37]	0	0.019
ritonavir/water [37]	0.00006	−0.059
PVPVA64/water [44]	0	−0.156

**Table 6:** Calculated composition of polymer-rich and drug-rich phases upon LLPS at *eGT* during hydration of 5 wt%, 15 wt%, 20 wt%, and 40 wt% DL ASDs based on ternary phase diagram in (Figure 2b). The calculated corresponding *T*g of the polymer-rich and drug-rich phases are also given. The lower and upper half-filled circle symbols schematically refer to the corresponding polymer-rich (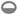) and drug-rich (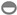) phases, respectively, after LLPS.

Ritonavir DL in the Dry ASD		Phase	Phase Mass Fraction at *eGT*	Phase Composition (Mass Fraction) at *eGT*			Phase *T*_g_ [°C]
				Water	Ritonavir	PVPVA64	
5 wt%	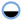	Polymer-rich phase	0.98	0.11	0.05	0.84	37.41
	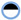	drug-rich phase	0.02	0.06	0.24	0.71	50.38
15 wt%	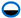	Polymer-rich phase	0.58	0.12	0.03	0.85	32.40
	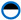	drug-rich phase	0.43	0.05	0.29	0.66	49.77
20 wt%	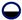	Polymer-rich phase	0.46	0.13	0.03	0.84	28.71
	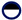	drug-rich phase	0.54	0.05	0.31	0.64	48.68
40 wt%	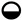	Polymer-rich phase	0.13	0.16	0.01	0.83	18.51
	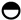	drug-rich phase	0.87	0.04	0.44	0.52	45.99

## Data Availability

The data presented in this study are available on request from the corresponding author due to confidentiality reasons.

## Supplementary Materials

The following supporting information can be downloaded at: https://www.mdpi.com/article/10.3390/pharmaceutics16101292/s1, Figure S1. Radii of gyration of the largest polymer/API clusters obtained from DPD simulations of the early stage of ASD dissolution, showing the disaggregation of the polymer/API present in the ASD (whose average size is indicated by the largest peaks in the plot) into smaller clusters at the concentration of 5 wt% (black bars) and 15 wt% (gray bars) at a simulation time of 1.4 μs; Figure S2. Radial density plots of copovidone (green lines) and ritonavir (black lines) within the main API/polymer cluster obtained during the late-stage dissolution molecular simulations for API concentrations of 1 wt% (a), 5 wt% (b), 15 wt% (c), 20 wt% (d), and 40 wt% (e).

## Abbreviations

The following abbreviations are used in this manuscript:

a	Helmholtz energy
AA	All-atom
A_i_,B_i_	Association sites A and B of molecule
bij	Equilibrium bond length
API	Active pharmaceutical ingredient
ASD	Amorphous solid dispersion
assoc	Association
${\Delta c}_{p,i}^{SL}$	Difference in solid and liquid heat capacity
DPD	Dissipative particle dynamics
DL	Drug load
disp	Dispersion
eGT	Escape glass transition
FTIR	Fourier-transform infrared spectroscopy
h_1+X_	Enthalpy
hc	Hard-chain
${\Delta h}_{i}^{SL}$	Melting enthalpy
K	Gordon-Taylor interaction parameter
k_B_	Boltzmann constant
k_ij_	Binary interaction parameter
L	Liquid
LLPS	Liquid-liquid phase separation
LoR	Loss of Release
LJ	Lennard-Jones
m	Mass
MD	Molecular dynamics
METT	Microscopic Erosion Time Testing
m_seg_	Segment number
*M*_w_	Molecular weight
*N*^assoc^	Number of association sites
NPT	Thermal-isobaric (constant number of particles, pressure, and temperature)
NVT	Thermal-isochoric (constant number of particles, volume, and temperature)
PC-SAFT	Perturbed-Chain Statistical Associating Fluid Theory
PVPVA64	Poly (vinylpyrrolidone-co-vinylacetate)
*R*	Universal gas constant
res	Residual
*R*_g_	Radius of gyration
RIT	Ritonavir
SLE	Solid liquid equilibrium
*T*	Temperature
*T*_g_	Glass transition temperature
$T_{i}^{SL}$	Melting temperature
u/k_B_	Dispersion-energy parameter
VCM	Vacuum compression molding
*w*	Mass fraction
*x*	Mole fraction
γ	Activity coefficient
ε_AiBi_/k_B_	Association-energy parameter
κ_AiBi_	Association-volume parameter
ρ	Density
θ_ij_	Equilibrium angle
σ	Segment diameter
