# Supplementary material for: Predicting the Release Mechanism of Amorphous Solid Dispersions: A Combination of Thermodynamic Modeling and In Silico Molecular Simulation
Source: Pharmaceutics. 2024 Oct 2;16(10):1292. doi: 10.3390/pharmaceutics16101292 (PMC11510624; doi:10.3390/pharmaceutics16101292)
Supplement: Supplementary file 1 [file pharmaceutics-16-01292-s001.zip › pharmaceutics-3182935-supplementary.pdf]

Supplementary Materials: Predicting the Release Mechanism of Amorphous Solid Dispersions: A Combination of Thermodynamic Modeling and in silico Molecular Simulation

Stefanie Walter[1], Paulo G. M. Mileo[2], Mohammad Atif Faiz Afzal[3], Samuel O. Kyeremateng[1*], Matthias Degenhardt[1], Andrea R. Browning[3], John C. Shelley[4*]

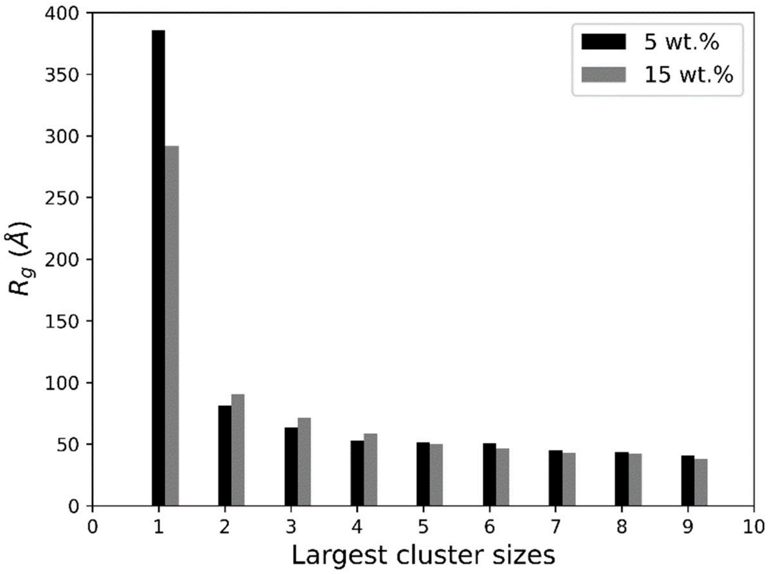

Figure S1. Radii of gyration of the largest polymer/API clusters obtained from DPD simulations of the early stage of ASD dissolution, showing the disaggregation of the polymer/API present in the ASD (whose average size is indicated by the largest peaks in the plot) into smaller clusters at the concentration of 5 wt% (black bars) and 15 wt% (gray bars) at a simulation time of 1.4 μs.

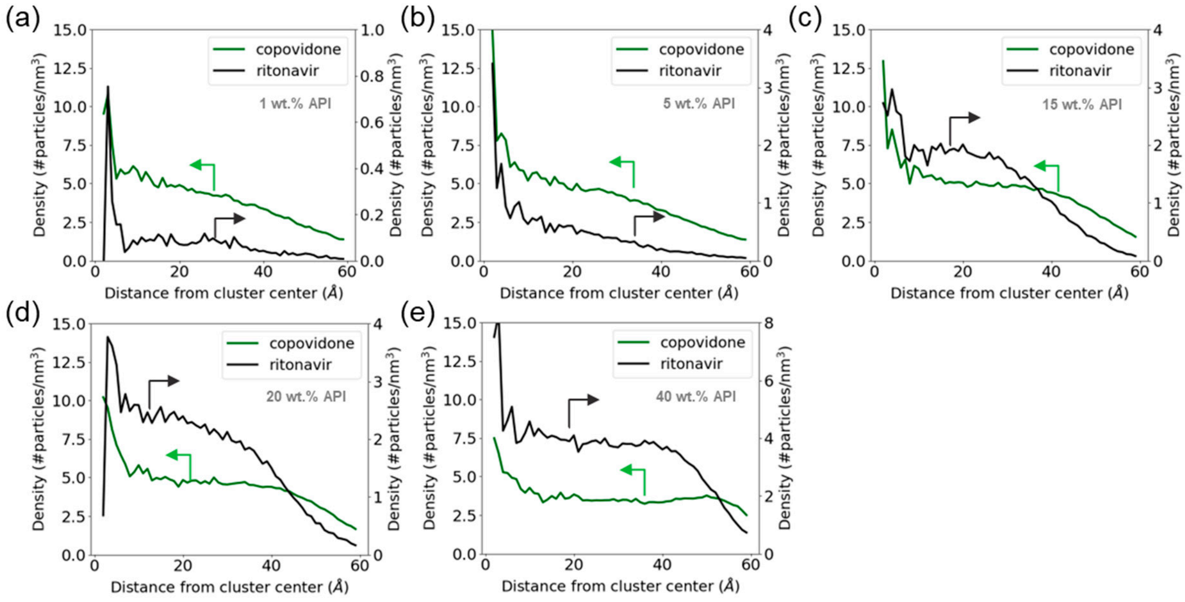

Figure S2. Radial density plots of copovidone (green lines) and ritonavir (black lines) within the main API/polymer cluster obtained during the late-stage dissolution molecular simulations for API concentrations of 1 wt% (a), 5 wt% (b), 15 wt% (c), 20 wt% (d), and 40 wt% (e).
